# Assessment of deltamethrin-induced DNA damage, neurotoxic and neuroimmune effects in the brain tissue of brown trout (*Salmo trutta fario*)

**DOI:** 10.17221/115/2023-VETMED

**Published:** 2024-03-28

**Authors:** Tayfun Karatas, Murteza Cakir

**Affiliations:** ^1^Health Services Vocational School, Agri Ibrahim Cecen University, Agri, Turkiye; ^2^Department of Neurosurgery, Medical Faculty, Ataturk University, Erzurum, Turkiye

**Keywords:** deltamethrin, DNA damage, fish, immunity

## Abstract

This study investigated the impact of deltamethrin (DM) toxicity on brown trout (*Salmo trutta fario*), examining its effects on the immune system, including the white blood cell (WBC), lymphocyte (Lym), total immunoglobulin (T. Ig), and lysozyme levels, as well as its neurotoxic consequences on the brain tissue. The neurotoxic effects encompassed oxidative stress, the activity of the antioxidant enzymes, such as the superoxide dismutase (SOD) and catalase (CAT), acetylcholinesterase (AChE) activity, and DNA damage using 8-hydroxy-2-deoxyguanosine (8-OHdG). The DM exposure led to elevated levels of malondialdehyde (MDA), and 8-OHdG, while concurrently causing a reduction in the AChE activity, protein and lipid content, WBC count, Lym, lysozyme activity, T. Ig levels, as well as the SOD and CAT levels in the brain tissues of groups 2 and 3 when compared to those in group 1. In summary, the findings of this study strongly indicate that DM induces DNA damage, immunotoxicity, and neurotoxicity in the brain tissue of brown trout, primarily due to the excessive production of reactive oxygen species (ROS). Moreover, the observed dose-dependent responses of DM to the environmental concentrations on all the investigated parameters suggest its potential utility in aquaculture risk assessment.

Pollution, including heavy metals, pesticides, sewage effluents, and petroleum, is a major threat to aquatic environments (Kong et al. 2021), with pesticides specifically posing risks to aquatic life due to their toxicity (Karatas et al. 2019). Pyrethroids, widely used for their high efficacy and low toxicity (Kong et al. 2021), can be more toxic than organophosphates due to their limited hydrolytic enzymes and heightened sensitivity in organisms (Arslan et al. 2017). These lipophilic compounds enter the body through fish gills, circulate in tissues, and accumulate, leading to toxicity in fish (Clasen et al. 2018). Fish exhibit high sensitivity to DM, a type II synthetic pyrethroid, in laboratory settings (Karatas et al. 2019). DM has a relatively short half-life in surface waters, rapid evaporation, and low persistence in pyrethroid resistance. Recent measurements have indicated that DM concentrations in surface waters can range from 2 ng/l to 4 μg**/**l (Lei et al. 2022).

It has been reported that deltamethrin may cause an increase in algae due to its effects on aquatic herbivorous insects (Amin and Hashem 2012). Pyrethroids, including DM, significantly slow the fish metabolism, exerting toxic effects on aquatic organisms at levels up to 10–1 000 times (Paul and Simonin 2006; Kong et al. 2021). Additionally, these pesticides exhibit lower degradation rates compared to those in birds and mammals (Kong et al. 2021) and can disrupt energy metabolism and ionic balance even at low concentrations like DM (Karatas et al. 2019).

This study was conducted to examine the impact of deltamethrin toxicity on the acetylcholinesterase (AChE) activity, oxidative stress, immune responses, and the level of 8-OHdG in the brain tissues of brown trout (*Salmo trutta fario*).

## MATERIAL AND METHODS

### Ethical approval

This study was performed within the ethical rules determined by Agri Ibrahim Cecen University (Writing and decision No.: 42162/132).

### Test organisms

Brown trout, each weighing an average of 80** ± **2 g, were acquired from the Faculty of Fisheries. The fish were divided into three groups, each comprising eight individuals, and each group was allocated to three individual tanks for a 15-day acclimation period (two replicates). Throughout the experiment, the fish were fed three times a day. The water used in the experiment was maintained at specific conditions, with a temperature of 9.6 ± 0.5 °C, dissolved oxygen levels of 9.5 mg/l, and a pH of 7.6.

### Acute toxicity

Deltamethrin (DM) with a purity of ≥ 98%, identified by CAS No. 52918-63-5, was procured from Sigma-Aldrich in Germany. The preparation of the stock solutions involved dissolving DM at a concentration of 5 mg/ml in a 1 : 1 mixture of ethanol and dimethyl sulfoxide (DMSO) (Karatas et al. 2019). In this study, 20% (0.8 μg/l) and 40% (1.6 μg/l) of the 4 μg/l dose reported by Lei et al. (2022) for surface waters of deltamethrin were used.

Group 1 represented the control, group 2 was subjected to 20% (0.8 μg/l) of DM, and group 3 was subjected to 40% (1.6 μg/l) of DM. The experiment was conducted with two replicates, and each group was monitored for a duration of fourteen days.

### Immunological analysis

The serum lysozyme enzyme was measured by a turbidimetric assay using *Micrococcus lysodeikticus* as described by Ellis (1990) (Sigma-Aldrich, St. Louis, MO, USA). The total immunoglobulin (T. Ig) levels in the fish were assessed following the procedure described by Siwicki (1993). The lymphocyte (Lym) and white blood cell (WBC) counts were determined using a Sysmex XN9500 modular system (Karatas et al. 2023).

### Malondialdehyde (MDA), antioxidant enzyme (SOD and CAT) proteins and lipid analysis

After removing the brain tissue, lipid extraction was carried out following the procedure described by Folch et al. (1957). Briefly, brain samples from the fish were homogenised in a mixture of chloroform–methanol (2 : 1, v/v) and then centrifuged (3 000 × *g*) for 10 minutes. In total, a 0.88% KCl solution was added to the supernatants and stored overnight at 4 °C. The chloroform-lipid phase taken with a syringe was transferred to a glass tube. Chloroform was evaporated with N_2_ gas for 3 h at a temperature of 40 °C in the glass tube (Karatas et al. 2021). The protein concentration in the brain tissues was quantified at 650 nm using the Lowry method, employing bovine serum albumin as a standard reference (Lowry et al. 1951). The MDA levels were determined at 532 nm, following the protocol outlined by Placer et al. (1966). The superoxide dismutase (SOD) activity was measured at 560 nm (Sun et al. 1988) and the catalase (CAT) activity at 405 nm (Goth 1991).

### Determination of the AChE activity

The brain tissues were homogenised by centrifugation at 3 500 x *g* for 10 min in a 0.05 M phosphate buffer solution, as described by Onalan and Yeltekin (2021). The acetylcholinesterase activity was assessed using the method established by Ellman et al. (1961). In this method, a mixture composed of the phosphate buffer, homogenate, 0.01 M dithionitrobenzoic acid (DTNB), and 1.25 M acetylthiocholine iodide was prepared. The resulting mixture was then spectrophotometrically measured at 412 nm over a period of 4–6 minutes. The protein concentrations in the brain tissue were determined following the procedure outlined by Lowry et al. (1951).

### Determination of the 8-hydroxy-2-deoxyguanosine (8-OHdG) level

The quantification of the 8-hydroxy-2-deoxyguanosine (8-OHdG) (Catalogue No.: 201-00-0041/SunRed) level in each fish’s brain tissue was performed utilising ELISA (enzyme-linked immunosorbent assay) kits in accordance with the instructions provided by the manufacturer, as specified by Kirici (2022).

### Statistical analysis

The data normal distribution (Shapiro–Wilk) and homogeneity of treatment variances (Levene test) were confirmed before analysis Subsequently, a one-way ANOVA (analysis of variance), coupled with Duncan’s post hoc comparison tests, was used to evaluate the significance of the biochemical and enzymatic data, with *P* < 0.05 considered statistically significant.

The statistical analysis of the data was carried out using SPSS v13.0 statistical software.

## RESULTS

### Effects of DM on the protein and lipid levels

**Figure 1:**
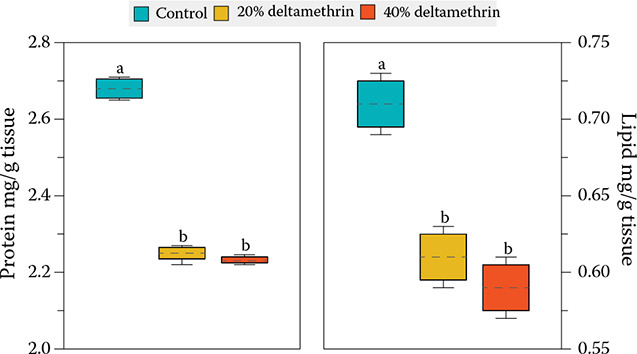
Influence of DM on the protein and lipid levels in the brown trout’s brain tissue Dashed lines indicate the mean values

Compared to group 1, exposure to DM led to a significant reduction in the protein and lipid levels in the brain tissues of groups 2 and 3 (*P* < 0.05). However, both of the dose increases did not affect the protein and lipid levels (*P* > 0.05) (Figure 1).

### Effects of DM on the oxidative stress and AChE activity

DM exposure led to a significant increase in the MDA levels and a simultaneous reduction in the antioxidant enzyme activities (SOD and CAT) and AChE levels in the brain tissues of groups 2 and 3 in comparison with group 1 (*P* < 0.05 (Table 1).

**Table 1:** Influence of the oxidative stress and AChE activity on the DM toxicity in the brown trout’s brain tissues

Parameters	Group 1	Group 2	Group 3
AChE (EU/mg protein)	1.01 ± 0.02^a^	0.81 ± 0.03^b^	0.93 ± 0.02^c^
MDA (mmol/g tissue)	62.9 ± 0.34^a^	80.5 ± 0.70^b^	70.2 ± 0.16^c^
SOD (EU/g protein)	58.4 ± 1.16^a^	46.9 ± 0.24^b^	51.3 ± 0.50^c^
CAT (kU/g tissue)	29.7 ± 1.52^a^	19.7 ± 0.51^b^	25.4 ± 0.85^c^

Data are presented as mean and standard deviation; Various symbols indicate the differences between the groups (*P* < 0.05); Group 1 was the control, group 2 was treated with 20% DM, and group 3 was treated with 40% DM

AChE = acetylcholinesterase; CAT = catalase; DM = deltamethrin; MDA = malondialdehyde; SOD = superoxide dismutase

### Effects of DM on the immunity

The WBC, T. Ig, lysozyme, and lymphocyte levels in groups 2 and 3 exposed to DM showed dose-dependent decreases, with statistical significance at *P* < 0.05 compared to group 1. However, there was no statistically significant difference between the WBC and Lym levels of groups 2 and 3 (*P* > 0.05) (Table 2).

**Table 2:** Effects of DM toxicity on immunity in brown trout’s brain tissues

Immunity	Group 1	Group 2	Group 3
WBC (10^4^/mm^–3^)	63 ± 1.87^a^	59 ± 2.0^b^	57 ± 1.88^b^
Lym (10^3^/μl)	44 ± 1.22^a^	39 ± 0.70^b^	38 ± 1.58^b^
Lysozyme (IU/ml)	93.4 ± 1.08^a^	80.3 ± 0.71^c^	71.1 ± 2.39^b^
T. Ig (mg/ml)	2.92 ± 0.05^a^	2.57 ± 0.02^c^	2.27 ± 0.02^b^

Data are presented as mean and standard deviation; Various symbols show the differences between the groups (*P* < 0.05); Group 1 was the control, group 2 was treated with 20% DM, and group 3 was treated with 40% DM

DM = deltamethrin; Lym = lymphocyte; T. Ig = total immunoglobulin; WBC = white blood cell

### Effects of DM on the 8-hydroxy-2-deoxyguanosine (8-OHdG) level

**Figure 2:**
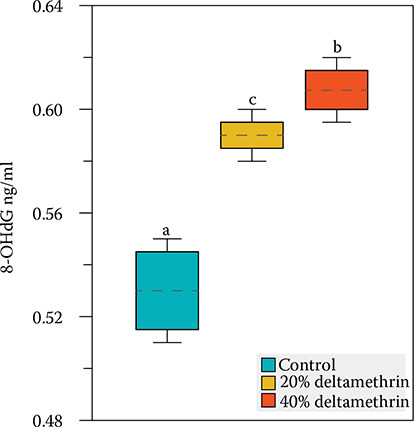
Influence of DM on the 8-OHdG level in the brown trout brain tissue Dashed lines indicate the mean values

The levels of 8-OHdG in groups 2 and 3 exposed to DM exhibited dose-dependent increases, with statistical significance at *P *< 0.05 in comparison to group 1 (Figure 2).

## DISCUSSION

AChE, which plays a key role in breaking down the neurotransmitter acetylcholine into choline and acetate, can be a key factor in detecting the effects of harmful substances (Schmidel et al. 2014). Topal et al. (2017) reported that pesticides that inhibit AChE can impair nerve function and lead to the excessive accumulation of acetylcholine (ACh) (Bhattacharya 1993). The decrease in AChE activity in groups 2 and 3 may result in the build-up of acetylcholine in the synapses, consequently disrupting various physiological functions (Glusczak et al. 2006; Karatas et al. 2019). Furthermore, the decline in AChE activity can contribute to neurotoxic alterations in the nerve function due to the reduced AchE expression (Da Cuna et al. 2011; Xing et al. 2013). Previous studies have demonstrated that pesticides induce a reduction in AChE activity within fish tissues (Topal et al. 2017; Karatas et al. 2019).

Oxidative stress develops when there is an imbalance between the production of free radicals and the protective mechanisms of antioxidants within the body (Li et al. 2011). ROS, which can result in molecular and cellular changes including DNA and antioxidant damage, have the potential to induce necrosis through the disruption of various physiological processes (Karatas et al. 2019). Furthermore, ROS have been identified as a factor that can impair organ functions by increasing cell membrane permeability (Mossa et al. 2013). Oxidative damage is recognised as a significant aetiological factor in neurodegenerative damage or disease (Hogg 1998). The observed elevation in levels of MDA, which is an indicator of oxidative stress, within the brain tissue of both DM-exposed groups confirms excessive free radical production. This phenomenon is likely attributable to the increased formation of reactive oxygen metabolites, particularly hydroxyl radicals. These radicals interact with phospholipid polyunsaturated fatty acids (PUFAs) in cell membranes, leading to the formation of unstable lipid peroxides that subsequently decompose into products such as MDA (Ogaly et al. 2015). Both doses of DM resulted in a decrease in the SOD and CAT levels within the brain tissues of groups 2 and 3. The decreased CAT activity in the brain tissues of DM-treated groups 2 and 3 may be associated with the increased production of oxidants and superoxide radicals (Karatas et al. 2019). Consistent with previous research, DM exposure has been shown to significantly reduce the antioxidant levels (Abdel-Daim et al. 2013; Ben Halima et al. 2014; Ogaly et al. 2015).

Proteins, which constitute the basic building blocks of living organisms, are accepted as the last class of biomolecules in terms of energy utilisation (Mishra et al. 2018). A significant decrease in the protein levels of DM-treated groups 2 and 3 was observed. This phenomenon may be a result of the inhibition of the translation process due to the increase in the rate of protein metabolism entering the Krebs cycle due to deltamethrin stress (Dubey et al. 2016).

Lipids, essential biochemical components, play a vital role by providing substantial energy through beta oxidation during oxidation and serving as structural elements for reproduction (Sargent 1995; Dunning et al. 2014). The lipid levels of DM-treated groups 2 and 3 were decreased compared to the control group. This may be a consequence of the oxidation of lipids to meet the increased energy demands of organisms due to the energy depletion caused by deltamethrin stress (Mishra et al. 2018).

Innate immunity, which encompasses elements, such as lysozyme, immunoglobulin, cytokines, transfer factors, complements, and lymphocytes, plays a pivotal role in the initial defence and autoimmunity of fish. This serves as the first line of defence against both pesticides and pathogenic microorganisms (Gou et al. 2018; Kong et al. 2021). The lysozyme, T. Ig, WBC, and Lym levels were significantly decreased in DM-treated groups 2 and 3 compared to group 1. The decline in protein content may, in part, be attributed to the reduction in the WBC levels, as WBCs are the main source of protein production, including lysozyme and immunoglobulin (Soltanian and Fereidouni 2017). Previous studies have proposed that the reduction in leukocyte production could be attributed to a significant deterioration or the chronic and possibly permanent suppression of non-specific immunity (El-Sayed and Saad 2008; Zhang et al. 2020). Our results are consistent with the findings of serum or plasma lysozyme and immunoglobulin assessments in various fish species, including *Gobiocypris rarus* (Zhang et al. 2020), *Oreochromis niloticus* (Dawood et al. 2020), *Sparus aurata* (Guardiola et al. 2014), and *Oncorhynchus mykiss* (Siwicki et al. 2010), when exposed to DM.

The increase in the 8-OHdG levels in the brain tissues of groups 2 and 3 exposed to DM may be a reactive response to the oxidative stress (Anjana Vaman et al. 2013). Onouchi et al. (2012) showed that the formation of superoxide anions (O_2_) and the subsequent oxidative stress can lead to an elevation in the 8-OHdG levels (Karatas et al. 2019). Studies involving DM exposure have consistently reported a significant increase in 8-OHdG levels (Arslan et al. 2017; Karatas et al. 2019).

The findings of this study show that environmental concentrations of DM cause a decrease in the antioxidant capacity and immune parameters, and an increase in the oxidative stress and 8-OHdG levels in the brain tissue of brown trout. Furthermore, the dose-dependent responses to environmental concentrations of deltamethrin across the assessed parameters provide valuable insights into its suitability for inclusion in aquaculture risk assessment protocols.
